# Incoherent Neutron Scattering and Terahertz Time-Domain Spectroscopy on Protein and Hydration Water

**DOI:** 10.3390/life13020318

**Published:** 2023-01-23

**Authors:** Hiroshi Nakagawa, Naoki Yamamoto

**Affiliations:** 1 Materials Sciences Research Center, Japan Atomic Energy Agency, Tokai-mura 319-1195, Ibaraki, Japan; 2 J-PARC Center, Japan Atomic Energy Agency, Tokai-mura 319-1195, Ibaraki, Japan; 3 Division of Biophysics, Department of Physiology, Jichi Medical University, Shimotsuke 329-0498, Tochigi, Japan

**Keywords:** incoherent neutron scattering, terahertz time-domain spectroscopy, correlation function, protein dynamics, hydration water dynamics

## Abstract

Incoherent inelastic and quasi-elastic neutron scattering (INS) and terahertz time-domain spectroscopy (THz-TDS) are spectroscopy methods that directly detect molecular dynamics, with an overlap in the measured energy regions of each method. Due to the different characteristics of their probes (i.e., neutron and light), the information obtained and the sample conditions suitable for each method differ. In this review, we introduce the differences in the quantum beam properties of the two methods and their associated advantages and disadvantages in molecular spectroscopy. Neutrons are scattered via interaction with nuclei; one characteristic of neutron scattering is a large incoherent scattering cross-section of a hydrogen atom. INS records the auto-correlation functions of atomic positions. By using the difference in neutron scattering cross-sections of isotopes in multi-component systems, some molecules can be selectively observed. In contrast, THz-TDS observes the cross-correlation function of dipole moments. In water-containing biomolecular samples, the absorption of water molecules is particularly large. While INS requires large-scale experimental facilities, such as accelerators and nuclear reactors, THz-TDS can be performed at the laboratory level. In the analysis of water molecule dynamics, INS is primarily sensitive to translational diffusion motion, while THz-TDS observes rotational motion in the spectrum. The two techniques are complementary in many respects, and a combination of the two is very useful in analyzing the dynamics of biomolecules and hydration water.

## 1. Introduction

Biological cells are composed of approximately 80% water and contain various biomolecules that function in an aqueous environment. Water molecules found on the surface of proteins in living organisms, termed hydration water, display physical properties that differ from those of ordinary water [1]. Proteins fluctuate with hydration water due to thermal agitation, which is characterized by *k_B_T*, where *k_B_* is the Boltzmann constant and *T* is the absolute temperature. For proteins that function in an aqueous environment, water does not exist merely as a solvent, but also affects protein stability and enzymatic reactions via hydration. To better understand protein function, it is important to analyze both the structure and dynamics of the hydration water of proteins. Incoherent inelastic and quasi-elastic neutron scattering (INS) and terahertz time-domain spectroscopy (THz-TDS) are molecular spectroscopic methods that span the terahertz frequency range (meV energy region) and are effective methods for analyzing the thermal fluctuation of proteins and their hydration water.

INS analyzes the hydration, temperature, and pressure dependence of the low-vibrational excitation peak of a protein, known as the boson peak, which is observed in the THz region of the neutron spectrum [2,3,4]. This apparently featureless spectrum contains information on intrinsic protein dynamics that are sensitive to environmental conditions. INS has been used to elucidate how protein hydration water dynamics induce protein dynamical transitions, which are coupled with fluctuations in the hydrogen bonding network [5,6]. Furthermore, INS has also been used to detect changes in protein dynamics that are associated with the folding of denatured proteins [7,8]. Recently, INS has often been used to study the protein dynamics in solutions [9,10]. INS can also selectively analyze molecular motion using deuterated labels [11,12]. Thus, INS has become a powerful tool to study the dynamic properties of proteins; however, it requires an accelerator or neutron source in a research reactor, which remains a hurdle for many researchers.

THz-TDS is one of a few methods that can measure vibrational spectra across a large range from a few cm^−1^ to several hundred cm^−1^ with high sensitivity. Compared to FT-IR vibrational spectroscopy, which measures a higher frequency region and determines the vibrations of a molecule’s functional group, THz-TDS measures energy regions that are two orders of magnitude lower in energy, thus allowing the larger-amplitude motions of the effective mass to be observed. Specifically, it corresponds to the vibrational motion of the secondary and tertiary protein structures [13]. THz-TDS has also been used to detect the dynamical transitions [14,15] and observe the interchain modes of polypeptides [16]. In the glassy state, the boson peak is also visible as with inelastic neutron scattering [17]. Recently, the hysteresis of hydrated protein dynamics was identified by terahertz spectroscopy and neutron scattering as the freezing of hydration water [18,19]. Although molecular dynamics simulations can easily calculate the INS spectrum quantitatively [20,21], it is not as easy to calculate the THz spectrum [22]. A major advantage of THz-TDS over INS is that the experiment can be performed on a lab scale.

THz-TDS and INS have many points in common and complementarity. One terahertz corresponds to approximately 33 cm^−1^, which overlaps with the INS measurement region. Although they share the same energy region to be observed, the information obtained is different. Despite the fact that a combined analysis of both methods could be considered beneficial, so far, not much has been achieved in the study of biomolecules. This review will introduce the differences between the information obtained from INS and THz-TDS, as well as the advantages and disadvantages of each and the experimental practical aspects, and emphasize the merits of the combined analysis of both methods. A few biomolecular analyses combining both methods will be presented [17,23]. The combined analysis of INS and THz-TDS will be very useful for analyzing the dynamics of biomolecules and hydration water.

## 2. Incoherent Neutron Scattering

### 2.1. Theory of Incoherent Neutron Scattering

INS measures the scattering intensity (i.e., double differential scattering cross-section) for the change in momentum and energy of neutrons as a result of their interaction with a sample. Here, the total cross-section is defined as

(1) $$\sigma =4\pi \langle b^{2}\rangle$$

where *b* is the neutron scattering length of the atom. The total scattering cross-section can be divided into two parts: The coherent scattering portion (i.e., the average scattering length for each atom), where the scattering waves are in phase, and the incoherent scattering portion (i.e., the deviation from the average scattering length of each atom), where the phase is random, as follows:

(2) $$\sigma =\sigma_{coh}+\sigma_{inc}$$

(3) $$\sigma_{coh}=4\pi {\langle b\rangle }^{2}$$

(4) $$\sigma_{inc}=4\pi \left(\langle b^{2}\rangle -{\langle b\rangle }^{2}\right)$$

The scattering intensity is proportional to a double differential cross-section, which is the number of neutrons scattered within an energy range Δ*E* and momentum variation in a solid angle ΔΩ. According to van Hove’s theory, for a system composed of a single atomic species, the double differential scattering cross-section can be written using the magnitudes of the momentum vectors of the neutrons before and after scattering by the sample (i.e., *k_i_* and *k_f_*), as follows:

(5) $$\frac{d^{2}\sigma }{d\Omega dE}=\frac{k_{f}}{k_{i}}\left\{\frac{\sigma_{coh}}{4\pi }S_{coh}\left(Q,\omega \right)+\frac{\sigma_{inc}}{4\pi }S_{inc}\left(Q,\omega \right)\right\}$$

(6) $$S_{coh}\left(Q,\omega \right)=\frac{1}{2\pi }\overset{+\infty }{\underset{-\infty }{\int }}dt\exp\left(-i\omega t\right)\underset{\alpha ,\beta }{\sum }\langle \exp\left(-i\vec{Q}\cdot {\vec{R}}_{\alpha }\left(0\right)\right)\exp\left(-i\vec{Q}\cdot {\vec{R}}_{\beta }\left(t\right)\right)\rangle$$

(7) $$S_{inc}\left(Q,\omega \right)=\frac{1}{2\pi }\overset{+\infty }{\underset{-\infty }{\int }}dt\exp\left(-i\omega t\right)\underset{\alpha }{\sum }\langle \exp\left(-i\vec{Q}\cdot {\vec{R}}_{\alpha }\left(0\right)\right)\exp\left(-i\vec{Q}\cdot {\vec{R}}_{\alpha }\left(t\right)\right)\rangle$$

where *k_i_* and *k_f_* are the absolute values of initial and final wavevectors before and after scattering, respectively, with *k_i_* or *k_f_* = 2π/*λ_i_* or 2π/*λ_f_* where *λ_i_* or *λ_f_* is the neutron wavelength before and after scattering; *S_coh_* is the coherent dynamic structure factor; *S_inc_* is the incoherent dynamic structure factor; *Q* is the momentum change; and *Rα(t)* represents the position of atom α at time *t*. Coherent scattering provides information about the relative atomic positions (i.e., structure), whereas structure information is lost in incoherent scattering, but molecular motions can be analyzed via the auto-correlation function.

### 2.2. Analysis of Incoherent Neutron Scattering

**Figure 1:**
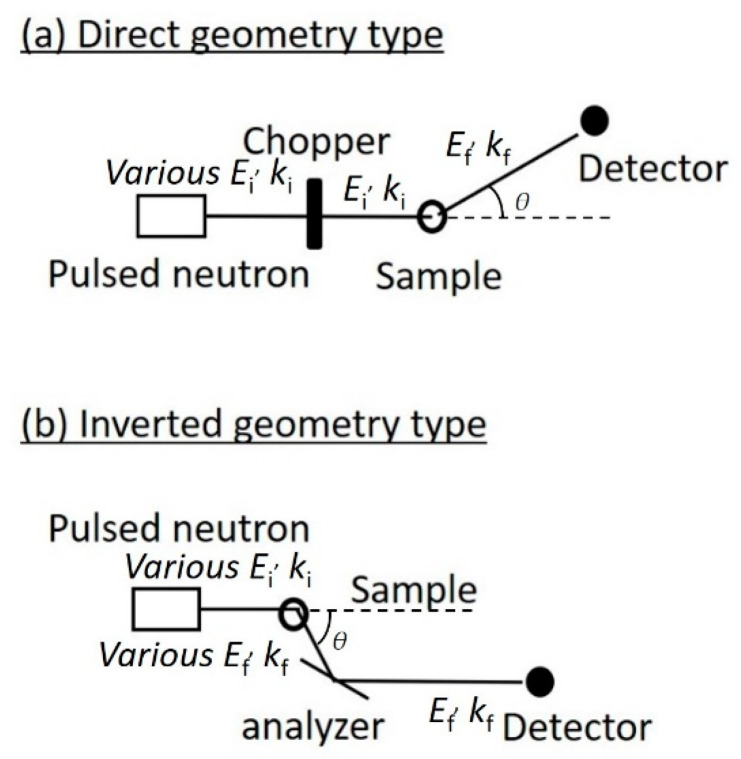
Conceptual diagrams of spectrometers installed at pulsed neutron sources: (**a**) Direct geometry and (**b**) inverted geometry types. The pulsed neutrons with various energy (*E*_i_) are produced at the neutron source. The neutron energy change, *ħω*, due to sample interactions, is given by the difference in the incident (*E_i_*) and final neutron energies (*E_f_*). The momentum change, *Q*, is calculated from initial and final wavevectors and the scattering angle that they form.

INS measures the energy changes between the incident and scattered neutrons and the momentum changes (*Q*), which do not involve optical processes. INS experiments measure neutron energy changes as a function of momentum change. The energy change is given by the difference in the incident (*E_i_*) and final neutron energies (*E_f_*): $\hbar \omega =E_{i}-E_{f}$, where *ω* is the angular frequency and ℏ is Planck’s constant. The kinetic energy is described as *E_i_ = mv_i_^2^/2* or *E_i_ = mv_f_^2^/2* where *m* is the neutron mass and *v_i_* or *v_f_* is the neutron velocity before or after scattering, respectively. The momentum change, *Q*, is given by:

(8) $$Q=\sqrt{k_{i}^{2}+k_{f}^{2}-2k_{i}k_{f}\cos\theta }$$

where *θ* is the scattering angle which the initial and final wavevectors form (Figure 1). The momentum change corresponds to the absolute value of the difference vector between the initial and final wavevectors.

There are various types of INS spectrometers that record $S_{inc}\left(Q,\omega \right)$. The three-axis spectrometer measures the intensity at each point in (*Q*, *ω*)-space that is physically accessible by the spectrometer. The time-of-flight (TOF) method can be used to explore rather large regions in (*Q*, *ω*)-space simultaneously and is often used for biological samples. There are two types of TOF spectrometers: Direct and inverted geometry. The conceptual diagrams for each type are shown in Figure 1. In direct geometry spectrometers, the incident pulsed neutron beam is monochromated by a chopper before interacting with a sample, where a single energy or wavelength is selected from the pulsed white beam using many different energies. The scattered neutron intensity is then measured as a function of the scattering angle and the time from the neutron source to the detector. In the inverted geometry spectrometer, the pulsed white neutron beam is scattered by interacting with the sample first, then the scattered neutrons are monochromated by an analyzer. Only neutrons with specific scattered energies are recorded by the detector.

### 2.3. Dynamic Information in Incoherent Neutron Scattering

**Figure 2:**
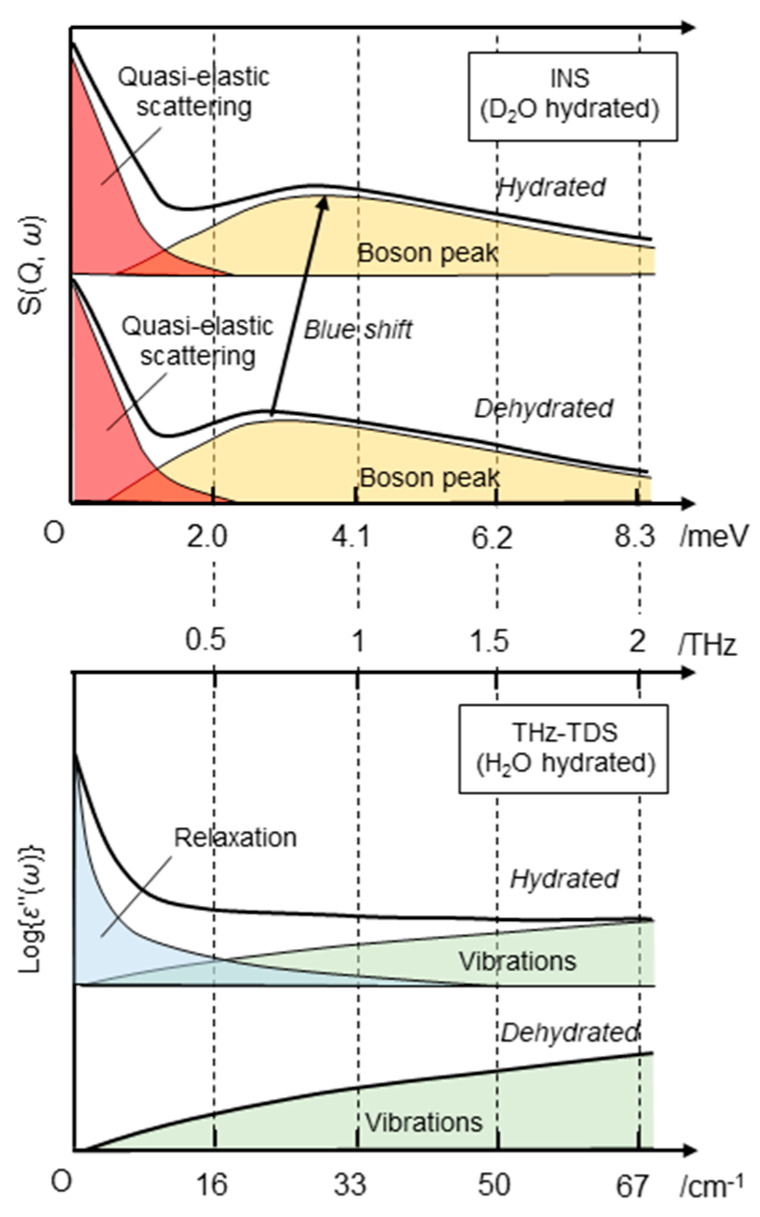
Schematic diagram comparing INS (**top**) and the imaginary portion of the complex dielectric spectra obtained by THz-TDS (**bottom**). The two spectra are displayed so that the energy units displayed on the vertical axes are aligned with their equivalent values. In the INS spectra, the contributions of quasi-elastic scattering and boson peaks are deconvoluted. Upon hydration, the top of the boson peak blue-shifts to the higher energy region. In the THz-TDS spectra, a relaxation mode appears upon hydration, which is deconvoluted from vibrations. Note that in the case of INS, a typical change hydrated with D_2_O is shown.

INS reveals the frequency distribution of molecular vibrations over a wide energy range of up to several hundred meV [2,3,4]. It has been suggested that the low vibration dynamics observed in the THz region (1 THz ≈ 4.1 meV ≈ 33 cm^−1^) are important for protein function [24]. The ability to measure this region accurately and quantitatively is a unique feature of INS compared to other spectroscopic techniques. INS spectra in the high-frequency region above 10 meV show little change due to hydration [3]; thus, hydration has little effect on the local dynamics of molecular groups, such as methyl groups [3,25]. In the region of a few meV, the change in the INS spectrum of a protein due to hydration is significant [2,3]. For example, a boson peak is observed at a few meV, which is common for synthetic polymers and glass-forming materials (Figure 2). Previous studies have shown that in the case of proteins, the boson peak position depends on the secondary structure [26] and molecular weight of the protein, indicating that it originates from the vibrational modes across the entire molecule [27]. Hydration causes the peak position to shift to higher energy, indicating that the protein structure becomes rigid upon hydration at low temperatures due to its hydrogen bonding with the hydration water [3] (Figure 2, top). When the protein structure fluctuates, the surrounding hydration structure should be simultaneously altered. The shift in the boson peak position indicates that the structural fluctuations throughout the protein are coupled with the hydration water [3]. Recent work has demonstrated that the peak position also depends on pressure [4]. Structural investigation of the effect of boson peaks on hydration, temperature, and pressure has revealed that the boson peak can be scaled by the cavity volume inside the protein [4]. Although the details of the molecular motion from which the Boson peak originates have not yet been fully elucidated, these studies have shown that the intensity and peak energy of the boson peak reflects intermolecular interactions and intra-molecular packing, and the structural state of a protein can be estimated using the boson peak as an indicator.

In recent years, INS research on proteins in a solution has become popular [10]. Since scattering from a solution sample includes a contribution from the solvent, the protein scattering can be measured as:

(9) $$S_{protein}\left(Q,\omega \right)=S_{solution}\left(Q,\omega \right)-\left(1-f_{ex}\right)S_{water}\left(Q,\omega \right)$$

where *S_protein_* and *S_solution_* represent the scattering factors of protein and solution, respectively; *f_ex_* is the fraction of the excluded protein volume in the solution, which is calculated from the solution concentration and the partial specific volume of the protein [7,9]. The dynamic structure factor of proteins in a solution includes contributions from the internal motion of the protein and the diffusion of the whole protein. Thus, the diffusion of the whole protein needs to be excluded to study the internal motion of the protein. In recent years, attention has been focused on the molecular crowding effect in cells [28]. In crowding environments where biological substances are present at high concentrations, it is important to measure the diffusion dynamics of proteins since the diffusion dynamics arer affected by the concentrated solution [29]. Although it is difficult to measure the diffusion coefficient of only the target molecule when multiple coexisting solute molecules are also present, the use of deuterated coexisting solute molecules makes it possible to measure the diffusion coefficient of the target protein with INS. Thus, INS should provide a unique method to characterize the diffusive motion of a specific biological material of interest on the spatial scale of the mesoscopic region at a time scale of pico- to nanoseconds.

There are various physicochemical measurement methods that study the dynamics of water in biological systems, such as protein hydration. For example, in dielectric spectroscopy, rotational motion can be determined from the response of the dipole moment of water molecules to an external electric field [18]. In contrast, the diffusive motion of water can be evaluated from a quasi-elastic neutron scattering in INS, which is described by the Lorentz function:

(10) $$L\left(\Gamma ,\omega \right)=\frac{\Gamma \left(Q\right)}{\pi \left[{\left\{\Gamma \left(Q\right)\right\}}^{2}+\omega^{2}\right]}$$

where $\Gamma \left(Q\right)$ is the half-width of the Lorentz function. In the small *Q* region, the translational diffusion coefficient, *D_T_*, can be obtained:

(11) $$\Gamma \left(Q\right)=D_{T}Q^{2}$$

In the *Q*-domain of approximately 1Å or more, the observed distance correlation becomes shorter, and the jump-diffusion behavior becomes apparent; therefore, the *Q*-dependence of the half-width can be calculated as:

(12) $$\Gamma \left(Q\right)=\frac{D_{T}Q^{2}}{1+D_{T}Q^{2}\tau }$$

where *τ* is the residence time of the water before the jump, and the half-width asymptotically approaches *τ = 1/Γ(Q)* at a higher *Q*.

### 2.4. Experimental Details

Proteins are biopolymers composed of amino acids. The atomic composition of proteins is composed of 50% hydrogen atoms. Thus, the incoherent scattering cross-section of hydrogen atoms is much larger than that of other elements, and the contribution from hydrogen atoms in INS is dominant in the total neutron scattering of proteins. Since hydrogen atoms in proteins are distributed almost uniformly in the three-dimensional structure, INS can be used to observe the average atomic fluctuations in a protein through the incoherent scattering of hydrogen atoms. Unlike coherent scattering, which needs to consider the time correlation between different atoms (i.e., pair-correlation function), incoherent scattering only needs to consider the time correlation of a single atom at different time intervals (i.e., auto-correlation function, Equation (7)). Thus, the molecular association and the resulting shape change of the molecule itself do not directly affect the spectrum. In addition, there is no need to align the orientation of the proteins in the sample for measurement, as is the case in crystal structure analysis, and any state (e.g., powder or solution) can be used for measurement. Furthermore, due to the high penetration of neutrons, the thermal fluctuations of molecules can even be measured in biological tissues, cells, and biomolecules in the pellet state. The fact that the experimental sample can be in any shape or state is advantageous and unique compared to other spectroscopic techniques.

When investigating the hydration effect on protein dynamics, it is convenient if the protein and hydration water dynamics can be observed separately. By taking advantage of the very different incoherent neutron scattering cross-sections of hydrogen and deuterium atoms, it is possible to separate the protein and hydration water [6]. If deuterated proteins are used, the scattering from the proteins can be minimized, and the hydration water dynamics can be analyzed more accurately [30]. This simple method of separating the scattering of protein and hydration water is based on the characteristics of incoherent scattering nature, in which only autocorrelation is observed. For example, small-angle scattering by coherent scattering has also been used to study protein hydration using the difference in scattering length between heavy water and light water [31]. However, it is not so simple to separate the structure and dynamics of each protein and hydration water in the coherent scattering of hydrated proteins because of the existence of cross-terms in the scattering function between the protein and hydration water [32].

Although commercial lab-scale instruments are available for THz-TDS, there are no small neutron sources that can provide a neutron beam of sufficient intensity. Thus, neutron-scattering experiments must be performed in large experimental facilities, such as research reactors and spallation source facilities. In a nuclear reactor, neutrons are generated by the fission reaction of uranium-235. In a spallation source facility, atoms in the target are spallated by colliding protons (H+) with a material called a target (e.g., mercury), and the resulting neutrons ejected from the target are used for experiments. To perform a neutron-scattering experiment, a proposal application describing the experimental plan must be submitted to each facility and pass an expert review panel. Since the application form requires specialized knowledge about scattering experiments, this requirement is a hurdle for researchers with no experience in neutron-scattering experiments.

## 3. Terahertz Time-Domain Spectroscopy

### 3.1. Theory of THz Spectroscopy

THz spectroscopy measures the absorption of the THz electromagnetic wave resulting from light–matter interactions. The interaction can originate from a molecular dipole moment or magnetism. The light–matter interactions in molecules that do not possess permanent molecular magnetism are reduced to an interaction between the light and electric dipole moment. In this situation, the Hamiltonian interaction between the light and dipole moment, *H*^(1)^(*t*), is described as:

(13) $$H^{\left(1\right)}\left(t\right)=-M\cdot E\left(t\right)$$

where **M** and **E***(t)* represent the molecular dipole moment and time-dependent electromagnetic wave, respectively. It is assumed that this interaction causes a transition from the initial state to the final state. According to Fermi’s golden rule, which is a perturbation theory formula of the time-dependent Schrödiner equation, a transition probability per unit time at the frequency of the electromagnetic wave *ω*, *P_i_*_→*f*_(*ω*), is described as:

(14) $$P_{i\to f}\left(\omega \right)=\frac{\pi E_{0}^{2}}{2\hbar^{2}}{\left|\langle f\left|\varepsilon \cdot Μ\right|i\rangle \right|}^{2}\left[\delta \left(\omega_{fi}-\omega \right)+\delta \left(\omega_{fi}+\omega \right)\right]$$

where *E*_0_ and **ɛ** represent the amplitude and unit vector in the direction of travel of the electromagnetic wave, respectively; $\langle f\left|\varepsilon \cdot M\right|i\rangle$ is the transition moment from the state *i* to *f* caused by the operator; ε·M. *ω_fi_* represents the energy difference between the initial and final state; and *ħ* is Planck’s constant. By considering all initial and final states and performing several mathematical calculations, an equation representing the absorption spectrum, *α*(ω), in the case of an isotropic material is finally obtained:

(15) $$\alpha \left(\omega \right)=\frac{2\pi \omega \left\{1-\exp\left(-\hbar \omega /k_{B}T\right)\right\}}{3\hbar cn\left(\omega \right)}\int_{-\infty }^{\infty }dt\exp\left(-i\omega t\right)\langle M\left(t\right)\cdot M\left(0\right)\rangle$$

where *n*(ω) is the index of refraction; $\langle M\left(t\right)\cdot M\left(0\right)\rangle$ is the time-correlation function of the total dipole moment of the system; and **M**(*t*). *k*_B_ and *T* represent the Boltzmann constant and absolute temperature, respectively. The integration is a line-shaped function because it determines the shape of the spectrum. Absorption spectra thus provide useful information on molecular dynamics.

Molecular motions in the sub-picosecond to picosecond range are monitored with THz spectroscopy, which overlaps the range monitored by INS. In contrast to INS, THz spectroscopy measures the total dipole moment of the system, which can be calculated as a sum of each dipole moment:

(16) $$M\left(t\right)=\underset{i}{\sum }\mu_{i}\left(t\right)$$

where $\mu_{i}\left(t\right)$ represents the *i*th dipole moment. Thus, $\langle M\left(t\right)\cdot M\left(0\right)\rangle$ is expanded as:

(17) $$\langle M\left(t\right)\cdot M\left(0\right)\rangle =\langle \underset{i\geq j}{\sum }\mu_{i}\left(t\right)\cdot \mu_{j}\left(0\right)\rangle$$

Assuming that each dipole moment behaves independently, the equation becomes:

(18) $$\langle M\left(t\right)\cdot M\left(0\right)\rangle =\underset{i}{\sum }\langle \mu_{i}\left(t\right)\cdot \mu_{i}\left(0\right)\rangle +\underset{i>j}{\sum }\langle \mu_{i}\left(t\right)\cdot \mu_{j}\left(0\right)\rangle$$

The first term on the right side of Equation (18) is an auto-correlation function of each dipole moment, which is in analogy to the quantity obtained by INS (i.e., auto-correlation of each atom as represented by Equation (7)). The second term on the right side of Equation (18) represents a cross-correlation of dipole moments, which is a quantity that is uniquely characteristic of THz-TDS and does not show up in INS. In this sense, THz spectroscopy and INS are similar but partially overlap. Thus, to strictly compare these two quantities, additional theoretical calculation methods are needed, such as molecular dynamics simulations.

### 3.2. THz-TDS Equipment

**Figure 3:**
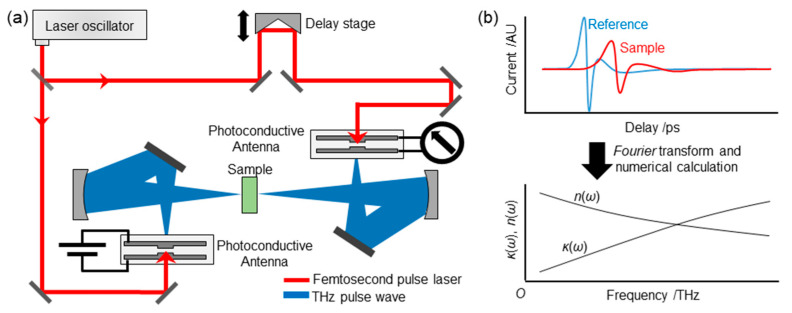
Schematic picture of (**a**) a THz-TDS setup and (**b**) a method to obtain the spectra of extinction coefficient and refractive index.

In THz-TDS, a THz pulse is generated, and the amplitude and phase of the pulse are detected in a time-resolved manner. A typical THz-TDS setup is shown in Figure 3a. To generate the THz pulse, a femtosecond pulse laser is typically used, where the pulse is split into two beams: One to generate and detect the THz pulse and the other for detection. One common technique to generate a THz pulse is to use a pair of photoconductive antennas, which have two metallic wires placed close together with a small gap (<1 mm) on a semiconductor substrate, and DC (direct current) bias is added between the wires in the antenna used for generation. When a femto-second pulse laser hits the gap, the electrons in the semiconductor are excited. The electrons are accelerated by DC bias, which causes the current. This current creates the pulsed electromagnetic wave in the THz frequency region. The THz pulse is then introduced to the other photoconductive antenna, which, in turn, further accelerates the electrons excited by the other femtosecond pulse, resulting in generating current. By varying the arrival time of the laser beam using a delay stage, the shape of the THz pulse can be directly monitored (Figure 3a,b, top).

### 3.3. Obtaining Spectra

In THz-TDS, a THz wave pulse in the time domain, E(*t*), interacts with a matter of interest, and the resulting reductions in amplitude and phase shifts are monitored. From these quantities, the extinction/absorption coefficient and refractive index can be calculated. Here, a method to obtain spectra is briefly introduced. A simplified drawing of how to calculate the spectra is shown in Figure 3b. An experimentally obtained THz pulse, *E*(*t*), is a Fourier transform of the electromagnetic wave in the frequency domain, *E*(*ω*):

(19) $$E\left(t\right)=\int_{-\infty }^{\infty }E\left(\omega \right)\exp\left(-i\omega t\right)d\omega$$

Thus, frequency-domain data can be obtained by performing a Fourier transform of *E*(*t*). Frequency-domain data after interacting with the sample, *E*_s_(*ω*), are described as follows:

(20) $$E_{s}\left(\omega \right)=E_{0}\left(\omega \right)\exp\left(ik_{s}\left(\omega \right)d\right)$$

where *E*_0_(*ω*) is the amplitude of the electromagnetic wave without the sample; *d* is the thickness of the sample; and *k*_s_(*ω*) is the complex refractive index. Hereafter, subscript *s* or *r* is used to represent a sample or reference, respectively. *k*_s_(*ω*) is a complex number composed of the refractive index, *n*_s_(*ω*), and the extinction coefficient, *κ*_s_ (*ω*):

(21) $$k_{s}\left(\omega \right)=\left(n_{s}\left(\omega \right)+i\kappa_{s}\left(\omega \right)\right)\frac{\omega }{c}$$

where *c* is the speed of light. Similarly, frequency-domain data without a sample (i.e., reference), *E*_r_(ω), are described as:

$$E_{r}\left(\omega \right)=E_{0}\left(\omega \right)\exp\left(ik_{r}\left(\omega \right)d\right)$$

where

(22) $$k_{r}\left(\omega \right)=\left(n_{r}\left(\omega \right)+i\kappa_{r}\left(\omega \right)\right)\frac{\omega }{c}$$

By measuring the ratio of the electromagnetic wave between the reference and sample, the sample spectrum is obtained by:

$$\begin{array}{l} \frac{E_{s}\left(\omega \right)}{E_{r}\left(\omega \right)}=\exp\left[i\left\{n_{S}\left(\omega \right)+i\kappa_{S}\left(\omega \right)\right\}\frac{\omega }{c}d-i\left\{n_{r}\left(\omega \right)+i\kappa_{r}\left(\omega \right)\right\}\frac{\omega }{c}d\right]\\ \equiv \exp\left\{-\kappa \left(\omega \right)\frac{\omega d}{c}\right\}\exp\left\{in\left(\omega \right)\frac{\omega d}{c}\right\} \end{array}$$

where

$$\kappa \left(\omega \right)=-\frac{c}{\omega d}ln\left(\left|\frac{E_{S}\left(\omega \right)}{E_{R}\left(\omega \right)}\right|\right)$$

and

(23) $$n\left(\omega \right)=-\frac{c}{\omega d}\arg\left(\frac{E_{S}\left(\omega \right)}{E_{R}\left(\omega \right)}\right)$$

The absorption coefficient is obtained from the extinction coefficient:

(24) $$\alpha \left(\omega \right)=\frac{2\omega }{\ln\left(10\right)}\kappa \left(\omega \right)$$

These quantities are convertible to the complex dielectric spectra,$\;\tilde{\varepsilon }\left(\omega \right)$:

$$\tilde{\varepsilon }\left(\omega \right)=\varepsilon^{\prime }\left(\omega \right)+i\varepsilon^{\prime \prime }\left(\omega \right)$$

where

(25) $$\begin{array}{cc} \varepsilon^{\prime }\left(\omega \right)= & n{\left(\omega \right)}^{2}+\kappa {\left(\omega \right)}^{2}\\ \varepsilon^{\prime \prime }\left(\omega \right)= & 2n\left(\omega \right)\kappa \left(\omega \right)=\frac{2c\alpha \left(\omega \right)n\left(\omega \right)}{2\omega } \end{array}$$

### 3.4. Experimental Details

Two methods are used for THz spectroscopy. One is transmission geometry, where a THz pulse passes through samples. The other is an attenuated total reflection (ATR) technique, where an evanescent THz wave generated on a THz transmissible crystal, such as silicon, is utilized. Here we focus on transmission geometry since ATR THz-TDS is described elsewhere [33].

In transmission geometry, a sample is vertically placed against the THz pulse. Liquid samples are installed into a spacer placed between two substrates, which is usually a silicon substrate with a thickness of ~5 mm. Solid samples (i.e., powders) are pelletized with a thickness of 0.1~1 mm depending on the degree of absorption of the THz waves. Pellets are usually placed in a sample holder to fix the sample position. The THz pulse is focused on the sample using mirrors (Figure 3a). Due to the diffraction of the THz wave, the pulse diameter at the focused point is in the sub-mm to mm order; thus, the diameter of the sample should be carefully determined. For example, the diameter should be approximately 1 mm at 1 THz (~33 cm^−1^) and 10 mm at 0.1 THz (~3.3 cm^−1^).

Temperature-dependent measurements are usually conducted for liquid or solid samples. In the case of liquid samples, a sample cell is set to a metallic holder at which the temperature is controlled using a coolant or Peltier temperature controller. In the case of a solid sample, the temperature points of interest cover cryogenic temperatures; thus, a cryostat is typically used. The cryostat windows usually consist of polyethylene, since it does not possess apparent absorption bands in the THz region.

Water is one of the most measured samples using THz-TDS. Despite its simple chemical structure, water possesses unique dynamics due to its hydrogen bonds. In addition, the absorption of water is large because of its large total dipole moment of the system, which originates from its hydrogen bond networks (Equation (15)). As a result, its sensitivity in THz spectroscopy is large. In the THz region, water possesses a fast rotational relaxation mode at ~30 cm^−1^, intermolecular vibrational modes at ~180 cm^−1^, and librational modes at ~600 cm^−1^, with a tail of a large rotational relaxation mode in the GHz region penetrating the THz region [34,35,36].

Due to the large absorption of bulk water, hydration water signals are usually eclipsed by those of bulk water. One way to extract information about hydration water in this situation is by monitoring the difference in the spectra with or without proteins. In this manner, Havenith and coworkers studied protein hydration water using THz spectroscopy [37]. They monitored differences in the absorption between protein solution and buffer solution at a limited spectral range and found a protein concentration dependence. They concluded that it is due to the overlap of the hydration shell around the protein.

A more direct method to monitor the effect of hydration water is adding a small amount of water or hydration. For example, protein dynamics affected by water have been monitored in this hydration-dependent manner. In such experiments, water molecules corresponding to the first or second hydration layers of proteins are added, and studies have typically focused on how these dynamics are affected by the solutes by measuring physical parameters, such as relaxation time and the frequencies of the vibrational modes, in a temperature-dependent manner.

For example, Markelz and colleagues studied the temperature and hydration dependence of various kinds of proteins in the THz region by changing the temperature from a cryogenic temperature (83 K) to room temperature [14,38,39]. Tominaga and co-workers extended the frequency region to GHz and monitored the temperature dependence of water and protein dynamics in a wide frequency range. They found that the relaxational mode in the GHz region still largely contributed to the hydrated protein at room temperature [18,19]; however, the relational component was still separable from the spectral components in the THz region, which allowed for protein dynamic information in the THz region to be obtained. A schematic drawing of the spectral changes upon hydration is described in Figure 2. In the dehydrated state, protein vibrational modes only exist in the THz region. Upon hydration, the relaxational component overlaps with the low-frequency side of the spectrum. This complex dielectric spectrum has been described as:

(26) $$\tilde{\varepsilon }\left(\omega \right)=\frac{\Delta \varepsilon }{1-{\left(i\omega \tau \right)}^{\alpha }}+\underset{k}{\sum }\frac{A_{k}}{\omega_{k}^{2}-\omega^{2}-i\omega \gamma_{k}}+\varepsilon_{\infty }$$

where the first term on the right side of the equation is the relaxational mode, which is described by a Cole–Cole type relaxation function; and Δ*ɛ*, *α*, and *τ* represent the dielectric amplitude, stretching parameter (0 < *α* < 1), and relaxation time, respectively (Figure 2, bottom “relaxation” in the hydrated state). The second term on the right side of the equation is the sum of the vibrational modes of the proteins and hydration water (Figure 2, bottom “vibrations” in the dehydrated and hydrated states). *A_k_*, *ω_k_*, and *γ_k_* represent the amplitude, center frequency, and damping constant of the *k*th vibrational mode, respectively. $\varepsilon_{\infty }$ corresponds to the dielectric constant of the high-frequency limit. The relaxational mode originates from rotational relaxations of hydration water that may couple with those of proteins. The vibrational modes are mixtures of fluctuations of hydrogen-bond networks of hydration water and protein low-frequency modes, which are delocalized in amino-acid residues or protein domains. These vibrational modes are usually overlapped and thus difficult to separate from each other. Separation of the relaxational mode from the vibrational mode is possible by obtaining complex dielectric spectra in the gigahertz (10^9^ Hz) region [18,19].

## 4. Computational Analysis

Molecular dynamics (MD) simulation is useful for studying protein dynamics. In recent years, significant progress has been made in computing hardware technology, and MD simulations have been actively used to study the structure and dynamics of biomolecules. In fact, MD simulations have been aggressively incorporated into the development of various experimental methods and the analysis of experimental data, including neutron scattering [6,11,21,30]. INS is compatible with MD simulations, which can investigate the pico- to nanosecond dynamics of molecules at the atomic level. In addition, INS can be directly compared to MD simulations [21] and serve as a guideline for simulation accuracy [20]. MD simulations allow us to visualize the molecular structure and dynamics at the atomic level. The dynamic structure factor can be easily calculated from the time-correlation of the atomic coordinates. Furthermore, experimental data can be quantitatively reproduced by considering the energy resolution of the apparatus, as shown in the following equation:

(27) $$S_{\mathrm{inc}}^{\exp}\left(Q,\;\omega \right)=R\left(\omega \right)⊗S_{\mathrm{inc}}^{\mathrm{MD}}\left(Q,\;\omega \right)$$

where $S_{\mathrm{inc}}^{\exp}$, $S_{\mathrm{inc}}^{\mathrm{MD}}$, and *R*(*ω*) represent the experimental dynamic structural factor, the dynamic structural factor calculated by MD simulation, and the energy resolution of the apparatus, respectively. A combined INS and MD simulation analysis is an effective method to analyze the structure and dynamics of biomolecules, since this combined approach analyzes the experimental data in a complementary and synergistic manner.

In contrast, THz-TDS is derived from the fluctuation of the dipole moment. Recently, terahertz spectra have been calculated via MD simulation [22], but so far, the direct comparison of THz-TDS and MD simulations is difficult. A simulation model that includes dipole effects needs to be developed before it can be directly compared to the experimental data. Since various cooperative motions exist in the THz region, it is necessary to construct an improved theoretical model to describe the experimental results more accurately. The development of a terahertz spectrum analysis method is expected to verify the validity of the theory and improve the theoretical model, which will greatly enhance protein dynamic analysis in the future.

## 5. Applications by Combined Analysis

### 5.1. Effect of Freezable and Unfreezable Hydration Water on Protein Dynamics

Protein dynamics are activated at ~220 K only when the protein is hydrated, which has been referred to as the protein dynamical transition [40]. This activation occurs after the dynamic coupling between protein and hydration water; however, the details of the coupling have not been fully understood. Recently, Yamamoto et al. found that some hydration water froze when the hydration degree was high using THz-TDS combined with a vector network analyzer that monitored the GHz region [18,19]. The authors then tried to elucidate how the freezable and unfreezable types of hydration water contributed to protein dynamic activation using neutron scattering [17]. As mentioned above, neutron scattering is one of the most powerful tools to study the effect of hydration on protein dynamics, because it can directly monitor protein dynamics by hydrating proteins with D_2_O instead of H_2_O. In the study, freezing was monitored by the *q*-dependent coherent scattering of ice, whereas the protein dynamics were evaluated using the energy-dependent incoherent scattering. A temperature hysteresis phenomenon in the diffraction peaks of ice from the freezable hydration water was observed, which was consistent with previous reports of THz-TDS or calorimetry [18,41]. In contrast, the protein dynamics did not display any temperature hysteresis. These results indicate that protein dynamics are not coupled with freezable hydration water dynamics. Instead, unfreezable hydration water is essential for the activation of protein dynamics. The study implies that the decoupling of the dynamics between unfreezable and freezable hydration water was the cause of the distinct contributions of hydration water to the protein dynamics. Hydration-coupled protein dynamics are characterized on a broad time scale and are hierarchical in time scale [42]. Although neutron scattering and terahertz scattering provide different information, the time scales of the observed phenomena themselves are common, and experimental information from both can be directly compared and discussed. By making effective use of such commonality and complementarity between INS and THz-TDS, this study elucidated the details of the interaction between proteins and hydration water that could not be clarified by only one of the two methods.

### 5.2. Hydration Water of Biocompatible Polymer

Another study that took advantage of the overlap of the time scales of these experimental techniques was performed by Tominaga et al. [23]. They evaluated the kind of water existing on a biocompatible polymer, poly(ethylene oxide). Using THz-TDS, they characterized bound water, solute-induced isolated water, and free water. On the other hand, using INS, they also confirmed that there is immobile, slow, and fast water. They concluded that even though THz-TDS and INS monitor different kinds of physical phenomena, i.e., rotational relaxations and diffusions, respectively, both could capture hydration water bound to the surface of the polymer, which could correspond to an “intermediate water” characteristic of biocompatible materials.

## 6. Concluding Remarks

In the United States, the SNS-pulsed neutron source is used for neutron scattering. In Japan, the J-PARC pulsed neutron source was completed in 2008 and is being upgraded in stages. The Laue–Langevin Research Institute in Grenoble (France) has the world’s highest power reactor (60 MW), and the European Spallation Source is under construction in Lund (Sweden), which is expected to be the world’s highest performance spallation source facility when it becomes operational. Although inelastic neutron scattering is a very powerful tool for studying protein dynamics, the annual neutron machine time available to a single researcher is very limited. In the X-ray research area, for example, laboratory-level X-ray diffractometers are available for preliminary experiments at large synchrotron radiation facilities. On the other hand, there are no equivalent laboratory-level instruments for INS experiments yet. Therefore, if there is a one-to-one or closer general relationship between INS and THz-TDS, then sufficient complementary terahertz measurements can be made before proceeding to neutron experiments. While laboratory-level X-ray diffractometers are available for preliminary experiments at large synchrotron radiation facilities, there are no equivalent laboratory-level instruments for INS experiments. Thus, THz-TDS will be an effective tool for the researchers concerned. Alternatively, if the hurdle to using neutrons can be lowered significantly for THz-TDS experts, then they will be motivated to use neutron facilities. It would be very beneficial for both parties if the research environment was established using THz-TDS and INS back-to-back. During the past 15 years, the development of THz-TDS has progressed, with many examples of its application to biological systems. With the development of terahertz wave generation technology, much research has been accumulated, including measurements of biomolecules, such as nucleobases, human cancer samples, and analyses of water dynamics in biological systems. These studies show that terahertz waves have the potential to become powerful tools in biophysics. Collaborative research in neutron, theoretical, and computational science based on terahertz spectroscopy will continue to become an important area of research in the future.

## Data Availability

Not applicable.
